# Three-Dimensional Simultaneous Arbitrary-Way Orbital Angular Momentum Generator Based on Transformation Optics

**DOI:** 10.1038/srep38667

**Published:** 2016-12-08

**Authors:** Chen Zhang, Li Deng, Wei Jun Hong, Wei Xiang Jiang, Jian Feng Zhu, Mi Zhou, Ling Wang, Shu Fang Li, Biao Peng

**Affiliations:** 1 Beijing Key Laboratory of Network System Architecture and Convergence, Beijing University of Posts and Telecommunications, Beijing 100876, China; 2 Beijing Laboratory of Advanced Information Network, University of Posts and Telecommunications, Beijing 100876, China; 3 State Key Laboratory of Millimeter Waves, Southeast University, Nanjing 210096, China

## Abstract

In wireless communications, people utilize the technology of diversity against multipath fading, so as to improve the reliability of communication equipment. One of the long-standing problems in diversity antennas is the limited number of diversity in a certain space. In this paper, we provide a solution to this issue by a three-dimensional (3D) simultaneous arbitrary-way orbital angular momentum (OAM) generator (3D SAWOG) based on transformation optics. The proposed 3D SAWOG consists of a metamaterial block and a group of transformation cylinders, by which arbitrary-way planar wavefronts can be converted to helical wavefronts with various topological charges simultaneously. The 2D four-way OAM generator and the 3D SAWOG are analyzed, designed, and simulated. The simulation results validate the performance of a 3D SAWOG successfully, indicating that the proposed model possess a high mode purity and expansibility. The SAWOG can be used as a novel diversity antenna array due to the orthogonal property among different modes, which could provide more degrees of freedom than traditional dual-polarization antennas, further improving the reliability of the communication systems.

As well known, the beam of orbital angular momentum (OAM) refers to a beam possessing a helical wavefront, which depends on the phase factor *e*^−*ilϕ*^. In general, Laguerre-Gaussian (LG) beam^1^,^2^,^3^ is a typical case. OAM is an important technology, which can add an extra dimension of the polarization to the traditional communication system in time domain and frequency domain. By adjusting the number of topological charges, phase coding^4^ can be constructed, and can directly increase the capacity of the channel. Furthermore, OAM beam has also been widely used in a variety of applications, including microparticles control^5^,^6^, optical communication^5^,^7^,^8^,^9^,^10^,^11^,^12^, quantum communication^13^, digital spiral imaging^14^, optical data storage^15^, and biophysics^16^.

In order to achieve helical beams, many methods have been proposed and comprehensively analyzed. Mode converters for producing specific modes are proposed by Allen for the first time, indicating that the spiral phase plates^17^,^18^ (SPPs) can be used to generate a helical phase. To decrease the discrepancies of the fabrication tolerances, a new method of holograms^19^ is then proposed, and the function of which is similar to the mechanism of optical diffraction gratings. However, the conversion efficiency and complexity of the computer hologram both are needed to be further improved. More recently, graphene patch reflectarrays^20^ and metasurfaces^21^ have also been designed as new methods to produce an OAM-carrying beam with single mode. The graphene patch reflectarray consisting of graphene patches with specific reflection coefficients is firstly proposed, providing a novel method to realize OAM^20^. The adjustable characteristics of graphene also provide a great flexibility to control the topological modes. In addition, the metasurface^21^ with circular slits possesses the high mode purity over a 2.7 THz bandwidth except for the deteriorated valley. These work have made huge contributions to the field of OAM, however, none of them proposed a practical plan for how to simultaneously generate arbitrary multi-mode OAMs. This kind of OAM generator has a potential value in wireless communication systems. The combination of the simultaneous arbitrary-way OAM generator (SAOG) and reflectors can be used as a novel diversity antenna array, which improves the number of the antenna diversity compared with traditional antenna arrays. The use of OAM antennas can improve the isolation due to its orthogonal property of different modes. It facilitates significantly shorting the distance of adjacent receiving antennas and reducing the overall size of the receiving device. Therefore, the study of the SAOG is meaningful and valuable for practical applications.

In this paper, based on the theory of transformation optics, a 3D simultaneous arbitrary-way orbital angular momentum generator is proposed for the first time to achieve OAM-carrying beams with various topological charges at the same time, as shown in Fig. 1. Firstly, a 2D four-way OAM generator with a variable emitting source is proposed, which can be split into arbitrary paths by employing coordinate transformations. Then, a 3D OAM generator with different topological charges on corresponding paths has been designed. The simulations of electric field and phase in different situations are presented to validate the generator’s performance. The proposed generator shows competitive advantages, including arbitrary-way generation of OAM, simultaneous different topological charges, and simple design procedure.

## Results

### Theoretical design

For a Gaussian-enveloped beam carrying an orbital angular momentum, its field is an eigenmode of the paraxial Helmholtz equation^1^,^2^,^3^,^22^,


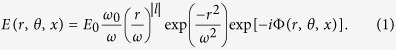


where *E*_0_ is the amplitude of electric field, ω_0_ is the waist radius. In addition, 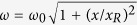, 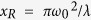, 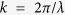, and *λ* refers to the beam width, Rayleigh length, wavenumber and wavelength in vacuum, respectively. The phase can be expressed as following^22^


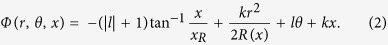


where 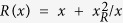 is the wavefront radius of curvature. It is the phase for 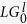 beam. In particular, this beam will degenerate into the Gaussian beam when *l* = 0. In order to generate OAM, it is important to manipulate phase and generate a helical wavefront *e*^*−ilϕ*^.

The key to generating OAM by transformation optics is to accurately set up the coordinate transformation of phase. The Jacobian matrix *A* depicts mapping relations between virtual space and physical:


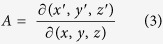


The relative permittivity *ε*′_*ij*_ and permeability *μ*′_ij_ tensors of transformation media are defined as^23^:


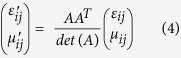


**Figure 2:**
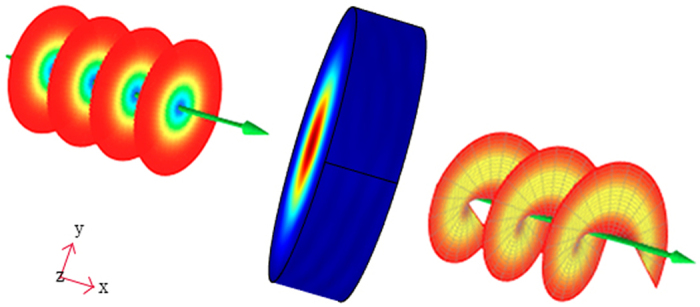
Conversion of a planar wavefront to a helical wavefront. A planar wavefront is an input along the axis x, then a helical wavefront will be achieved when it go through the transformation cylinder.

According to equations (3)–(4), constructive parameters of the metamaterial cylinders could be calculated. The parameters obtained could effectively manipulate the wavefront in the transformation cylinder so as to generate a beam with OAM. Through this transformation cylinder, Gaussian beam propagating along axis x will be converted to a helical Laguerre-Gaussian beam with a phase *e*^*−ilϕ*^, as shown in Fig. 2.

### Numerical calculation

In the simulation, the Gaussian beam is employed to propagate along the symmetrical axis x through the metamaterial cylinder whose central axis is also parallel to the axis x. Parameters *a, r*, and *l* denotes the thickness, radius, and the topological charge, respectively. A proportional coefficient *n* must satisfy the following relation:


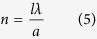


This relation is the prerequisite of Gaussian beam’s transformation in the range of cylinder. In next part, some simulations performed by the multi-physics simulation tool (COMSOL) will be presented to reveal the physical process and confirm the generation of OAM.

Based on the principle of transformation optics, a transformation from physical space (*x, y, z*) to the virtual space (*x*′*, y*′*, z*′) is defined as following:


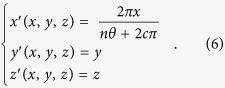


where *n* is the proportional coefficient, and *c* is a constant related to initial point coordinate so as to avoid the singularity in the calculation. In addition, 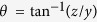 is the azimuthal angle in the *yoz* plane of the transformation cylinder and range of *θ* is from 0 to *2π*. Then, the Jacobian matrix *A*^24^ according to equation (3) can be calculated:


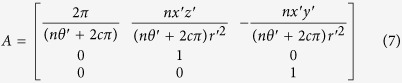


where 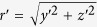. Generally, relative permittivity and permeability *ε*_*ij*_ and *μ*_*ij*_ both equal to 1 in free space. Therefore, the relative permittivity and permeability of transformation media^24^ can be acquired using equation (4):


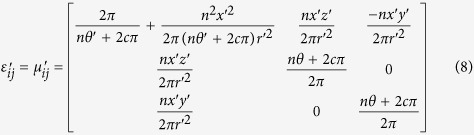


From the above, the wavefront transformation is *ndθ/2π* and the longitudinal phase shift is *ndθ/λ* at the position with azimuthal angle *θ*. Therefore, the OAM *L* generated in the x-direction is


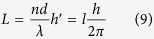


where *h* is the Planck’s constant. Considering the property of OAM, the phase difference is *lλθ/2π* and the helical wavefront is *e*^*−ilϕ*^.

### 2D four-way OAM generator (FWOG)

**Figure 3:**
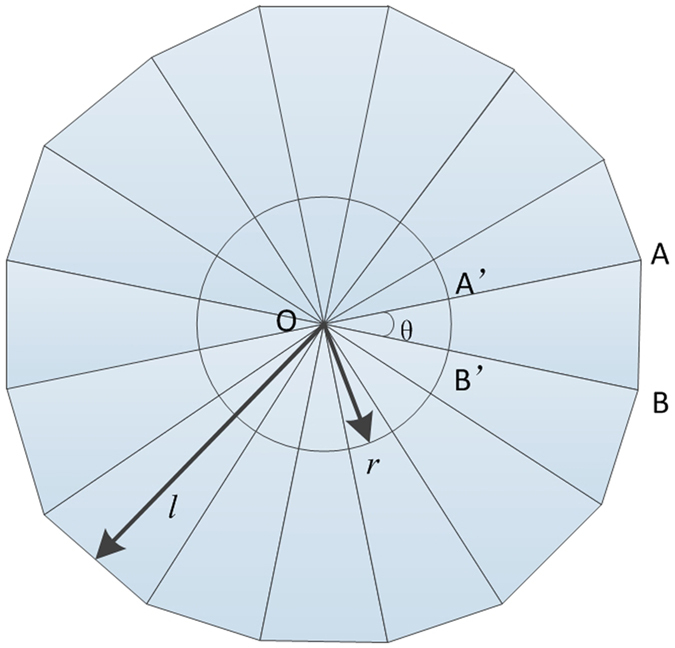
The scheme of one beam splitting into N beams.

In our proposed scheme, the central metamaterial square can split a source into arbitrary beams. Some methods have been proposed to support the realization of this scheme. A general method^25^ is employed here, as Fig. 3. The virtual space OA′B′(x, y, z) is mapped to physical space OAB (x′, y′, z′) with transformation function as:


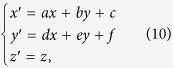


and


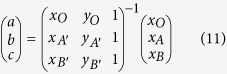


Constitutive tensors of material in the transformation region can be obtained:


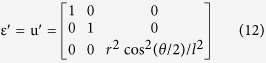


where *r* is a radius of the circle, and *l* is the vertical distance from the source to a side of the polygon, and the θ is the central angle of a circle. The radiation direction varies with the relative position of points (A, A′, B, B′). For the TE incident wave, we can simplify the parameters as following:


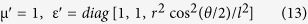


This method results in generally arbitrary emission direction for an individual beam. In addition, this design can be fabricated with copper strips and dielectric substrates by current techniques^25^. Therefore, the scheme that splits one beam into arbitrary beams is viable in the practical applications.

**Figure 4:**
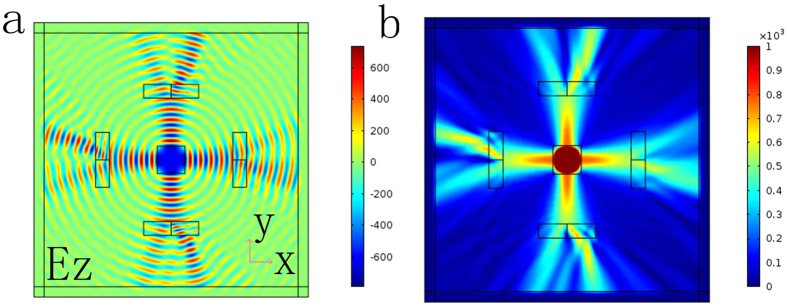
Results of the 2D FWOG. **(a)** Electric field distributions of the 2D FWOG. **(b)** Power flow distributions of the 2D FWOG. The z-component of the electric field and power flow both operate at 4 GHz. For a rectangle, width is 2*λ* and the length is 4*λ*. The Gaussian beam located at the center is divided into four directions as the input beams. Each rectangle contains two sections, the azimuthal angle of one section is set as 0 and another is *π*/2.

In order to verify the results of the 2D FWOG, simulations are carried on based on the method of finite elements (i.e., Comsol). Considering the performance of computer and complexity, a 2D FWOG is firstly presented in Fig. 4. The constructive parameters can be obtained by equation (8) under the condition of *z* = 0. The Gaussian beam located at the center is divided into four directions to perform as the input for each transformation cylinder. Four rectangles represent the cylinders’ profiles with different topological charges, and the value of each cylinder increases from *l* = 1 to *l* = 4 anticlockwise.

In order to observe the change of the electric field in a 2D model, each rectangle is divided into two sections, the azimuthal angle of one section is set as 0 and another is *π/2*. As shown in Fig. 4, there is a significant difference between the electric field and power flow, confirming that the wavefronts of output beams have shifted at different azimuthal angles, which indicates the wavefront from an output surface would spirally propagate along the symmetrical axis x. The proposed generator can be further extended to an arbitrary-way generator by changing the angle θ, which is related to the number of splitting beams. Compared with the method to overlap more than one grating, the proposed method performs high convenience and flexibility, and can be put into practice based on transformation optics^26^,^27^,^28^,^29^,^30^.

### 3D multiway OAM generator (MWOG)

**Figure 5:**
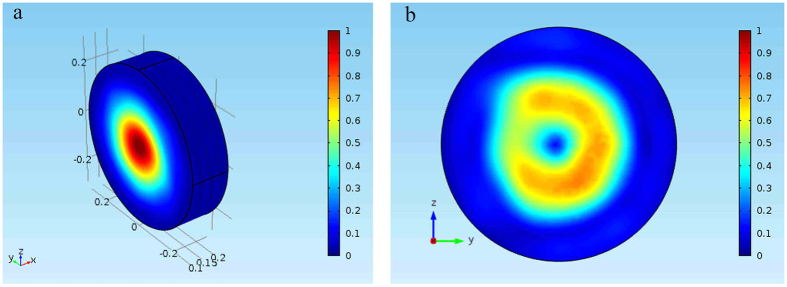
Model of 3D transformation cylinder. **(a)** 3D transformation cylinder in the coordinate. In this model, thickness *a* = 2*λ*, the radius of cylinder *r* = 4*λ*, constant *c* = 1, wavelength, and waist radius of input Gaussian beam *ω* = 2*λ* are selected. **(b)** The intensity on the exit transverse section is zero at the central position, while it does not equal to zero around the center.

Compared with the 2D OAM simulations, a 3D MWOG provides a better way to show the characteristics of the helical electric field and phase, for which reason, a 3D transformation cylinder is built according to equation (8), as depicted in Fig. 5(a). In general, the intensity of an output beam is zero at the central position, while the intensity around center does not equal to zero because the output beam propagates along axis x in a spiral way. The simulated result observed from the yoz plane is shown in Fig. 5(b), i.e., a typical distribution for Laguerre-Gaussian beam when its topological charge is one.

Several reasons cause the lack of symmetry in the intensity maps. Firstly, the intensity of the orbital angular momentum that we observe is the average intensity over a period. In the simulation, the result that we get is the distribution at a certain time. Therefore, a diagram of power density may not be a standard ring shape. Secondly, the degree of mesh and proportional coefficient c both will influence final results. However, the former is the main reason.

**Figure 6:**
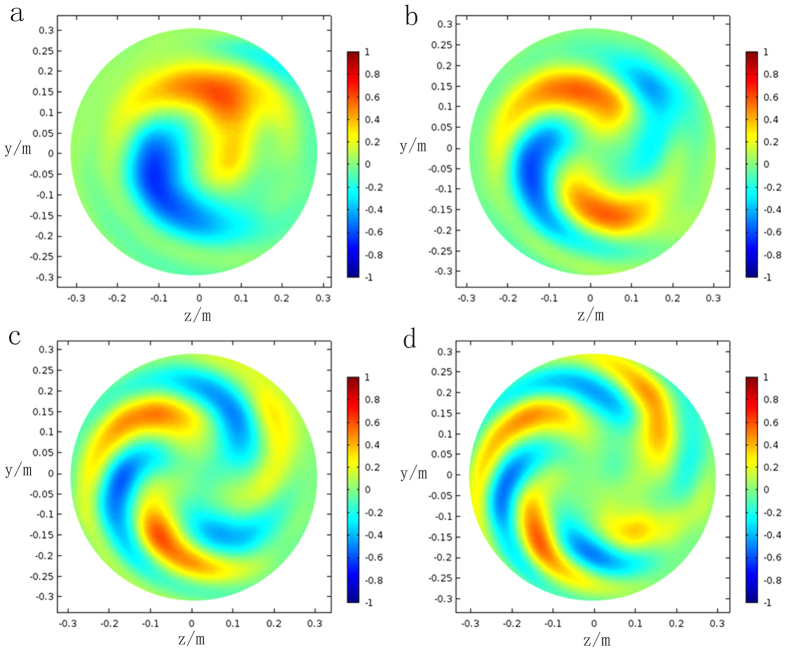
The electric field of the output beam from topological charges *l* = 1 to *l* = 4. The z component electric field of the exit surface in the situation *l* = 1 to *l* = 4 are depicted in the graph. These results are observed at the position 3*λ* away from the transformation cylinder’s exit surface **(a)** *l* = 1. There are two helical arms circling clockwise. **(b)** *l* = 2. **(c)** *l* = 3. **(d)** *l* = 4.

**Figure 7:**
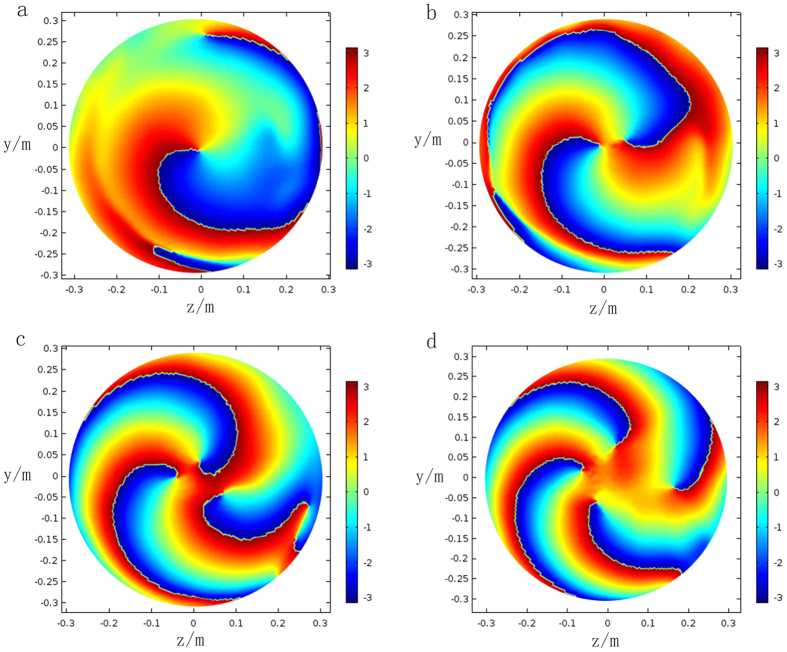
Phases of the output beam from topological charges *l* = 1 to *l* = 4. The phase results are observed at the position of 3*λ* away from the exit surface. The planar phases of input beams are converted to helical phases. **(a)** *l* = 1. The variation from blue color to red color corresponds to the phase change from −*π* to *π*. **(b)** *l* = 2. **(c)** *l* = 3. **(d)** *l* = 4.

In order to clearly display the output field and phases with different topological charges, results of the transverse section will be presented. The constructive parameters can be calculated by equations (7)–(9). The z-component electric field and phases of the exit surface in the situation *l* = 1 to *l* = 4 are depicted in Figs 6 and 7. Each transformation cylinder is excited by a Gaussian beam with the planar wavefront, and resultant electric fields and phases are observed at the position of 3*λ* away from the exit surface. In one hand, for the case of *l* = 1 in Fig. 6(a), two helical arms circling clockwise. Furthermore, the number of helical arms will also increase an additional two along with the topological charges gradually increasing. In the other hand, it clearly indicates that the phase gradually changes from −*π* (blue color) to *π* (red color) in Fig. 7(a), which experience a phase variation of 2*π* from anticlockwise direction. This result is consistent with equation (1) and proves that the wavefront of the output beam carries OAM in situation *l* = 1. The rest results of phase also verify this thought. In terms of relative knowledge of LG beam, a conclusion can be drawn that each output beam has successfully possessed an OAM of *L* = *lh*′.

## Discussion

In this paper, a 3D SAWOG has been proposed for the first time, by which, arbitrary beams with a variety of modes can be obtained simultaneously. The simulation results have verified the performance of a 3D SAWOG successfully, indicating that the proposed model possess a high mode purity and expansibility. The 3D SAWOG can be used as a novel diversity antenna array due to the orthogonal property among different modes, which would provide more degrees of freedom than traditional dual-polarization antennas, further improving the reliability of the communication systems. Furthermore, compared with traditional polarization diversity antennas, the proposed SAWOG can significantly reduce the distance between adjacent antennas. Due to the decreased distance between adjacent antennas, the SAWOG facilitates the miniaturization of the antenna arrays, which is very promising for future applications in the next generation wireless communication system^31^,^32^,^33^,^34^,^35^,^36^.

## Additional Information

**How to cite this article**: Zhang, C. *et al*. Three-Dimensional Simultaneous Arbitrary-Way Orbital Angular Momentum Generator Based on Transformation Optics. *Sci. Rep.* **6**, 38667; doi: 10.1038/srep38667 (2016).

**Publisher's note:** Springer Nature remains neutral with regard to jurisdictional claims in published maps and institutional affiliations.

## Figures and Tables

**Figure 1:**
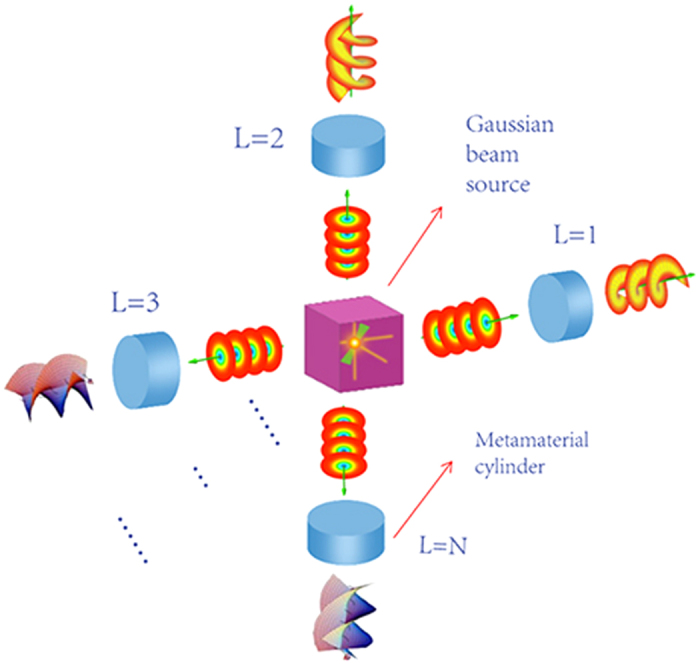
Diagram of the 3D simultaneous arbitrary-way OAM generator. In the central position, there is a square filled with metamaterials, which can split the Gaussian beam source into arbitrary ways of beams, so as to produce the input for each metamaterial cylinder. In addition, transformation cylinders with specific topological charges are placed outside the source. On the different paths, the input Gaussian beams will be converted into the LG beams with corresponding helical wave fronts and topological charges.
